# Alternating Electric Fields Modify the Function of Human Osteoblasts Growing on and in the Surroundings of Titanium Electrodes

**DOI:** 10.3390/ijms21186944

**Published:** 2020-09-22

**Authors:** Franziska Sahm, Josefin Ziebart, Anika Jonitz-Heincke, Doris Hansmann, Thomas Dauben, Rainer Bader

**Affiliations:** 1 Biomechanics and Implant Technology Research Laboratory, Department of Orthopaedics, Rostock University Medical Centre, 18057 Rostock, Germany; anika.jonitz-heincke@med.uni-rostock.de (A.J.-H.); doris.hansmann@med.uni-rostock.de (D.H.); rainer.bader@med.uni-rostock.de (R.B.); 2 Institute for Medical Microbiology, Virology and Hygiene, University Medical Center, Schillingallee 70, 18057 Rostock, Germany; thomas.dauben@uni-jena.de

**Keywords:** alternating current, electrical stimulation, electric field, osteoblasts, bone remodelling, osteoblasts differentiation factors, osteogenesis

## Abstract

Endogenous electric fields created in bone tissue as a response to mechanical loading are known to influence the activity and differentiation of bone and precursor cells. Thus, electrical stimulation offers an adjunct therapy option for the promotion of bone regeneration. Understanding the influence of electric fields on bone cell function and the identification of suitable electrical stimulation parameters are crucial for the clinical success of stimulation therapy. Therefore, we investigated the impact of alternating electric fields on human osteoblasts that were seeded on titanium electrodes, which delivered the electrical stimulation. Moreover, osteoblasts were seeded on collagen-coated coverslips near the electrodes, representing the bone stock surrounding the implant. Next, 0.2 V, 1.4 V, or 2.8 V were applied to the in vitro system with 20 Hz frequency. After one, three, and seven days, the osteoblast morphology and expression of osteogenic genes were analysed. The actin organisation, as well as the proliferation, were not affected by the electrical stimulation. Changes in the gene expression and protein accumulation after electrical stimulation were voltage-dependent. After three days, the osteogenic gene expression and alkaline phosphatase activity were up to 2.35-fold higher following the electrical stimulation with 0.2 V and 1.4 V on electrodes and coverslips compared to controls. Furthermore, collagen type I mRNA, as well as the amount of the C-terminal propeptide of collagen type I were increased after the stimulation with 0.2 V and 1.4 V, while the higher electrical stimulation with 2.8 V led to decreased levels, especially on the electrodes.

## 1. Introduction

The implantation of a total hip replacement is a common surgery which is being performed with increasing frequency due to the rising age of the global population [1]. Surgery is indicated in the case of trauma, osteoarthritis, avascular necrosis of the femoral head, or bone loss resulting from tumours [2,3,4,5]. In a study by Springer et al., it was observed that about 10% of all patients sustained a loosening of the implant in the 15-year period after the surgery [6]. Notably, 80% of these cases represent aseptic loosening, although there is strong evidence regarding the high percentage of false-negative microbiologic results due to the insensitivity of the method [7,8]. To avoid aseptic loosening, a promising option for the enhancement of early and stable implant fixation is the application of electrical stimulation as an adjunct therapy, during the critical phase after surgery. Bone formation and bone remodelling are known to be triggered by the application of mechanical loads on the bone tissue [9]. Since Fukada and Yasuda discovered that endogenous electrical fields arise in bone through the application of mechanical force, a lot of research and clinical applications have been done in the field of electrical stimulation [10]. The use of the inverse piezoelectric effect to improve bone formation by electrical stimulation (ES) is already utilised as an adjunct therapy with good levels of evidence for non-unions, ankle/foot unions, spinal fusions, and necrosis of the femoral head [11,12,13]. ES can be applied by direct, capacitive, or inductive coupling. Thereby, electric fields are delivered by direct or alternating current. Using a direct coupling system, electrodes and power supply are placed invasively on the affected bone and therefore can lead to the risk of infection, soft tissue irritation, and a second surgical procedure for electrode removal after bone healing/regeneration. Capacitive coupling, using electrodes attached to the skin above the bone defect, is non-invasive ES. However, skin irritation can occur during capacitive coupled electrical stimulation and the application of high voltages is necessary to induce appropriate electric fields inside the bone tissue. Inductive coupling delivers the pulsed electromagnetic fields (PEMFs) via one or two magnetic coils placed over the bone defect and therefore induce an electrical field through PEMFs while connected to an outer current supply [11]. Another option for the application of ES after joint arthroplasty is the integration of devices for electrical stimulation into the endoprosthetic implant. A possible design of such an implant has been described previously [14,15]. The advantages of this method are the possibility of direct electrical stimulation at the desired site of the bone regeneration and the need for low patient compliance.

The effect of direct current (DC) and PEMFs on osteoblasts and mesenchymal stem cells has been demonstrated in vitro in several studies, showing an increase in proliferation, differentiation factors like osteocalcin, osteopontin, and Runt-related transcription factor 2 (RunX2), an increased alkaline phosphatase (ALP) activity, and calcium deposition [16,17,18,19,20,21,22,23]. However, research on electric fields generated through alternating current (AC) is less common but interesting, since the application of AC reduces corrosion processes at the electrodes that contribute to particular wear generation and metal ions release on the implant surface [24]. Besides this advantage of AC, it leads to a reduction in the pH shifts and gas formation, which would otherwise lead to tissue irritation. In our previous work, we have established an in vitro system for the alternating current stimulation where bone cells can be cultivated on top of rough electrodes made of Ti6Al4V [25]. These represent the surface of an endoprosthetic implant that delivers ES. Thereby, osteoblasts were cultivated in small distances to the electrodes on collagen-coated glass coverslips in order to approach physiological growth conditions in an implant’s surroundings. Hence, the effects of AC stimulation on contact and distant osteogenesis could be investigated with the in vitro stimulation system. In the present study, the influence of different voltages resulting in varying electric fields were analysed in order to determine the suitable voltages for the application in AC stimulation.

## 2. Results

### 2.1. Influence of Alternating Electric Fields on Osteoblast Morphology

**Figure 1:**
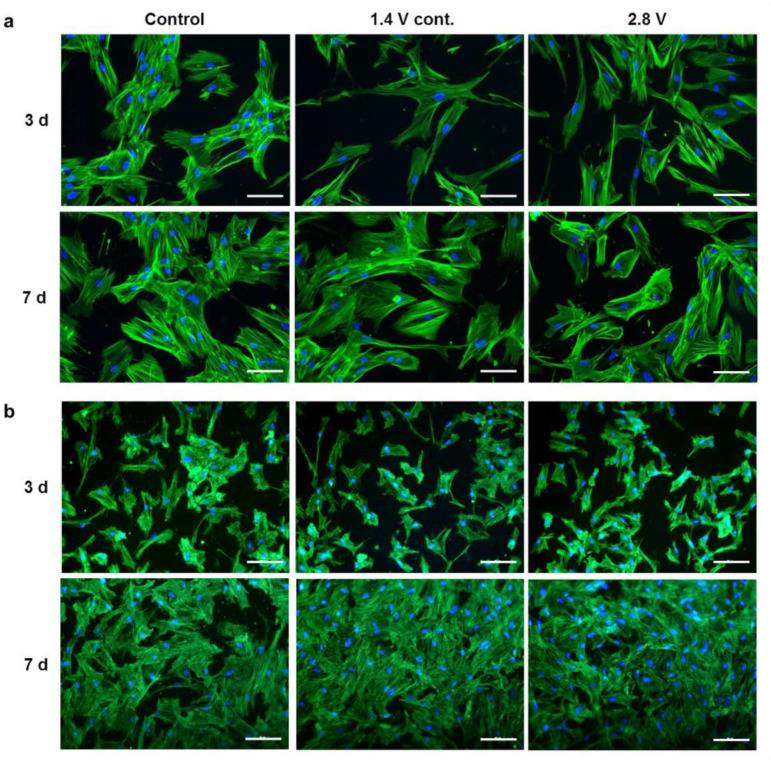
Actin staining of human osteoblasts on collagen-coated coverslips (**a**) and Ti6Al4V electrodes (**b**) in unstimulated cells, after electrical stimulation (ES) with 1.4 V cont. and 2.8 V after three and seven days. The 2.8 V group was stimulated 3 × 45 min per day, while the 1.4 V cont. group was stimulated continuously with 1.4 V. The scale measures 50 µm.

The viability of human osteoblasts, after their exposure to alternating electric fields for three and seven days, was ensured by actin staining following the strongest stimulation protocols, which were 1.4 V continuous and 2.8 V intermittent electrical stimulations (ES). The cell morphology, intensity, and orientation of actin fibres were unchanged after ES, compared to the unstimulated controls on collagen-coated coverslips (Figure 1a) and titanium electrodes (Figure 1b) at both time points. The dimensions of osteoblasts growing on rough Ti6Al4V electrodes were smaller than those growing on collagen-coated coverslips due to the rough surface. However, the osteoblasts were able to proliferate on the electrodes, showing a higher cell density after seven days, compared to the three-day time point, and formed a confluent cell layer on both the rough surface of the electrode and the smooth surfaces of the collagen-coated coverslip under control and ES conditions.

### 2.2. Effects of Alternating Electric Fields on Osteoblastic Proliferation

**Figure 2:**
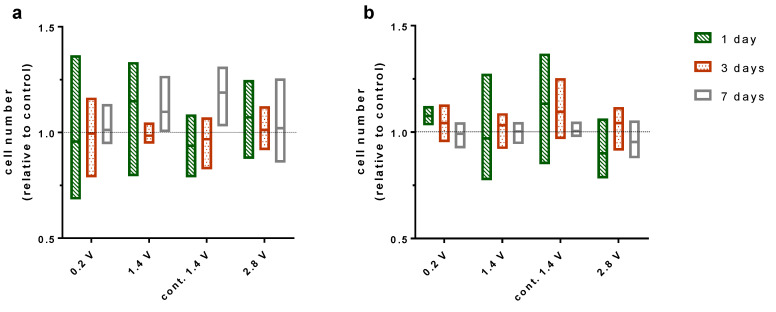
Cell number of osteoblasts cultured one, three, and seven days with and without electrical stimulation counted via ImageJ. Cell number is displayed relative to the control for each time point. For this experiment, 0.2V, 1.4 V, and 2.8 V groups were stimulated 3 × 45 min per day while the cont. 1.4 V group was stimulated continuously with 1.4 V. Cells grown on Ti6Al4V electrodes (**a**) and collagen-coated coverslips (**b**). *n* = 3.

Electrical stimulation showed no significant influence on the proliferation of human osteoblasts during seven days of stimulation using different applied electrical fields. Furthermore, the proliferation was not changed through the intensity of the electrical field acting on the cells. Cells cultivated on the electrode showed similar proliferation as cells cultured on collagen-coated coverslips on the bottom of the well (Figure 2).

### 2.3. Effects of Alternating Electric Fields on Osteogenic Gene Expression

Three genes for osteogenic differentiation, collagen 1 (*COL1A1*), alkaline phosphatase (*ALP*), and osteocalcin (*BGLAP*) were examined. *COL1A1* and *ALP* were expressed at every time point for each ES. *BGLAP* expression could be detected after three and seven days of cultivation.

**Figure 3:**
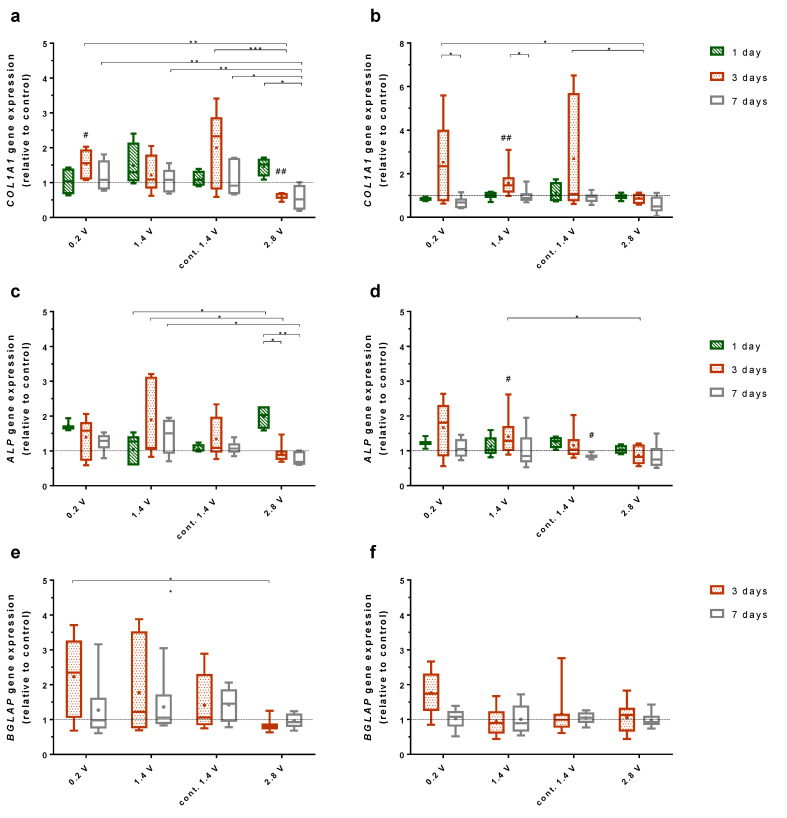
2^−ΔΔCt^ values for the gene expression of collagen type I (*COL1A1*; **a**,**b**), alkaline phosphatase (*ALP*; **c**,**d**), and osteocalcin (*BGLAP*; **e**,**f**) after electrical stimulation, compared to the unstimulated controls for the same time points. Cells were cultured on Ti6Al4V electrodes (**a**,**c**,**d**) and collagen-coated coverslips (**b**,**d**,**f**) for one, three, and seven days. The 0.2V, 1.4 V, and 2.8 V groups were stimulated 3 × 45 min per day, while the cont. 1.4 V group was stimulated continuously with 1.4 V. # indicates significant differences between the stimulated and control groups: # *p* < 0.05 and ## *p* < 0.01 (Wilcoxon matched pair test), * indicates significant differences between stimulation groups: * *p* <0.05, ** *p* < 0.01, *** *p* < 0.001 (two-way ANOVA). *n* ≥ 3.

*COL1A1* expression in human osteoblasts on Ti6Al4V electrodes was mainly downregulated through stimulation with 2.8 V over time. After one day of cultivation with 2.8 V, the gene expression was slightly upregulated but dropped significantly after seven days (*p* = 0.0190). Further, after three days, the gene expression was significantly downregulated compared to 0.2 V and 1.4 V continuous stimulation (*p* = 0.0044, *p* = 0.0004, respectively) and compared to the unstimulated control (*p* = 0.0078). The same progression could be observed for the cultivation with 2.8 V over seven days, as the gene expression for ES with 0.2 V, 1.4 V, and continuously 1.4 V was significantly higher compared to 2.8 V (*p* = 0.0089, *p* = 0.0027, *p* = 0.0299, respectively) (Figure 3a). The expression of *ALP* showed similarities to the *COL1A1* gene expression from cells grown on electrodes. Cells stimulated with 2.8 V expressed a higher amount of *ALP* after one day of stimulation but reduced their expression after three days (*p* = 0.0460) and seven days (*p* = 0.0061) of cultivation. Compared with the data from ES using 1.4 V, cells stimulated with 2.8 V expressed significantly less *ALP* after three days (*p* = 0.0178) and seven days (*p* = 0.0337) (Figure 3c). *BGLAP* expression of cells growing on electrodes was significantly lower after ES with 2.8 V compared to ES with 0.2 V after three days (*p* = 0.0166) (Figure 3e).

Cells growing on coverslips showed less change in the gene expression of *COL1A1, ALP*, and *BGLAP* after ES compared to cells growing on electrodes. *COL1A1* expression was increased after three days for ES with 0.2 V, 1.4 V, and continuous 1.4 V. Thereby, the increase after three days of stimulation with 1.4 V was significant (*p* = 0.0078). Furthermore, the decrease of the *COL1A1* expression from three to seven days was significant for 0.2 V and 1.4 V (*p* = 0.0369, *p* = 0.0218, respectively). Cells grown under ES with 2.8 V for three days expressed significantly less *COL1A1* compared to cells with 0.2 V (*p* = 0.0319) and continuous 1.4 V (*p* = 0.0386) (Figure b). As for the electrodes, cells growing on coverslips showed a similar expression behaviour of *ALP* and *COL1A1.* ES with 1.4 V led to a significant increase in *ALP* expression after three days compared to the unstimulated control (*p* = 0.0319) and the stimulation with 2.8 V (*p* = 0.0155). As for *COL1A1*, the expression of *ALP* decreases under ES with 2.8 V with time. Furthermore, a stimulation with continuous 1.4 V led to decreased *ALP* expression after seven days (*p* = 0.0313) (Figure 3d). No significant change of *BGLAP* expression could be observed for the cultivation under electrical stimulation (Figure 3f).

### 2.4. Effects of Alternating Electric Fields on Osteogenic Mediators

**Figure 4:**
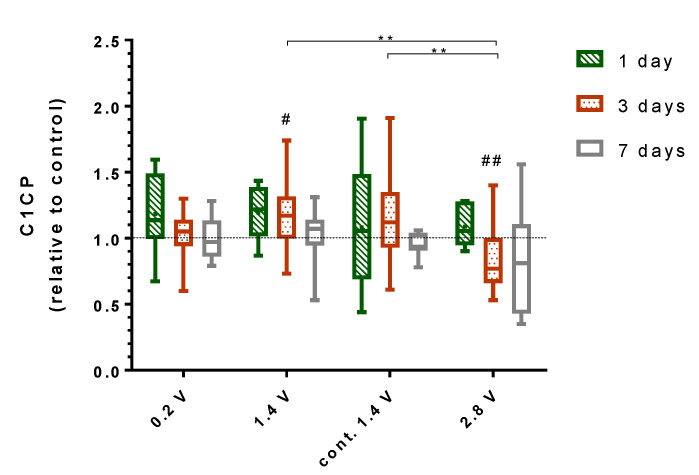
Collagen type 1 propeptide (C1CP) synthesised by human osteoblasts growing on electrodes and coverslips for one, three, and seven days. Peptide concentrations were determined from cell culture supernatant with and without electrical stimulation, and values are displayed relative to the unstimulated control for each time point. For this experiment, 0.2V, 1.4 V, and 2.8 V groups were stimulated 3 × 45 min per day, while the cont. 1.4 V group was stimulated continuously with 1.4 V. # indicates significant differences between the stimulated and control groups: # *p* < 0.05 and ## *p* < 0.01 (Wilcoxon matched pair test), * indicates significant differences between stimulation groups: ** *p* < 0.01 (two-way ANOVA). *n* ≥ 6.

De novo synthesis of collagen type I of the osteoblasts growing on both the electrodes and coverslips was evaluated by the detection of the propeptide of collagen type I (C1CP) in the cell culture supernatant. A significant increase in the C1CP synthesis was found after ES with 1.4 V (*p* = 0.0391), while 2.8 V stimulation significantly decreased the C1CP synthesis after three days of ES (*p* = 0.0059), as compared to the unstimulated controls (Figure 4). Differences between both stimulation groups were significant (*p* = 0.0056). Further, the amount of C1CP differed significantly between the stimulation with continuous 1.4 V and 2.8 V (*p* = 0.0095) after three days. No changes in the C1CP synthesis were detected after seven days of ES compared to the unstimulated cells or between the different stimulation groups.

**Figure 5:**
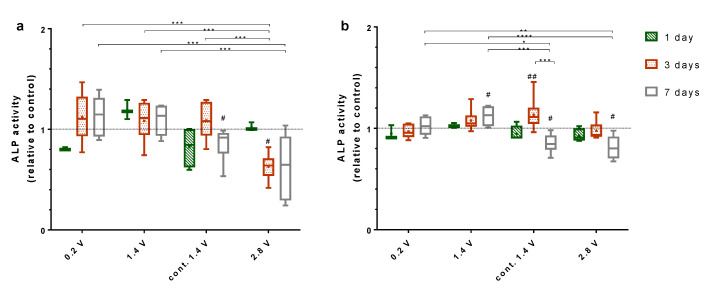
Alkaline phosphatase activity (ALP) of human osteoblasts growing on electrodes (**a**) and coverslips (**b**) with and without electrical stimulation. Results are displayed relative to the unstimulated controls after one, three, and seven days. For this experiment, 0.2 V, 1.4 V, and 2.8 V groups were stimulated 3 × 45 min per day, while the cont. 1.4 V group was stimulated continuously with 1.4 V. # indicates significant differences between the stimulated and control groups: # *p* < 0.05 and ## *p* < 0.01 (Wilcoxon matched pair test), * indicates significant differences between stimulation groups: * *p* < 0.05, ** *p* < 0.01, *** *p* < 0.001 and **** *p* < 0.0001 (two-way ANOVA). *n* ≥ 3.

The alkaline phosphatase (ALP) activity from cells growing on electrodes was significantly elevated after their exposure to 0.2 V, 1.4 V, and continuous 1.4 V compared to 2.8 V (*p* = 0.0004, *p* = 0.0003, *p* = 0.0002, respectively) (Figure 5a). After seven days of stimulation, this trend was continued for 0.2 V and 1.4 V compared to 2.8 V (*p* = 0.0002, *p* = 0.0005, respectively). Seven days after exposure to 1.4 V continuous stimulation, the ALP activity decreased significantly (*p* = 0.0313). Further, ES with 2.8 V reduced the ALP activity on the electrodes significantly after three days (*p* = 0.0313) and non-significantly after seven days.

Cells cultivated on collagen-coated coverslips with a stimulation of 0.2 V and 1.4 V showed increasing ALP activity over time (Figure 5b). After seven days, the ALP activity for ES with 1.4 V increased significantly compared to the control (*p* = 0.0313). Furthermore, the ES with 0.2 V and 1.4 V led to a significant rise in ALP activity after seven days compared to ES with continuous 1.4 V (*p* = 0.0276, *p* = 0.0001, respectively) and 2.8 V (*p* = 0.0044, *p* < 0.0001). Cells stimulated continuously with 1.4 V initially showed a significant increase in ALP activity after three days (*p* = 0.0078), though the ALP activity decreased significantly after seven days compared to three days (*p* = 0.0001) and the unstimulated control (*p* = 0.0313). A similar significant decrease could be observed for cells cultivated over seven days with 2.8 V ES (*p* = 0.0313).

## 3. Discussion

In the present in vitro study, we investigated the response of human osteoblasts to alternating electric fields when seeded directly on the rough surface of Ti6Al4V electrodes, modelling an implant surface equipped with an AC stimulator for bone cell growth on the implant. In these experiments, the osteoblasts were also seeded at a short distance to the electrodes on collagen-coated coverslips to evaluate the effects of electrostimulation on bone cells growing in the tissue surrounding the implant.

Staining the cytoskeleton of the osteoblasts with phalloidin showed no effect of electrical stimulation with 2.8 V or continuous stimulation with 1.4 V on actin organisation. The cells were able to maintain a spread phenotype and proliferate evenly on electrodes, demonstrating that the osteoblast viability was not deteriorated by the electrical stimulation applied in our study. Quantification of the stained nuclei of the osteoblasts could equally show that the amount of cells on the electrodes and coverslips was not reduced by ES.

Effects of ES on osteoblast functions were highly voltage-dependent. While 0.2 V and 1.4 V resulted in enhanced expression of the key mediators involved in bone formation (C1CP, and ALP), 2.8 V reduced the osteoblast function. Applying the same voltage, the continuous stimulation was not found to be superior to an intermittent stimulation (3 × 45 min per day), having even a negative effect on the expression of the investigated mediators, such as ALP after seven days, as compared to the unstimulated controls. Considering the required energy supply of the electrically active implants, shorter stimulation periods are also preferable over continuous stimulation. Compared to the three-day stimulation period, the positive effects of ES on the osteoblast function were slightly diminished after seven days of ES, as the C1CP concentration as well as the *COL1A1* gene expression dropped from three to seven days. On the other hand, ALP activity was constant or even rose over time. Su et al. showed that the gene and mediator expression of differentiation factors can vary over time when using capacitive ES [26]. Therefore, it would be interesting to cultivate the cells over a longer time period to survey possible fluctuations of the osteogenic markers under ES. Further, as various differentiation factors are expressed on different time points throughout osteoblasts differentiation, it is possible that other factors which were not examined were positive influenced through ES [27].

Although significant effects dependent on the applied AC voltage could be demonstrated in our study, these effects were relatively small. The sensitivity of human osteoblasts from different patients to ES was subjected to high variances. Since human osteoblasts were pre-differentiated during the cultivation before conducting the experiments, cells might have reached a high differentiation state, thereby further limiting the effects of ES. Therefore, the use of mesenchymal stem cells with a high differentiation capacity might elicit more pronounced effects from the applied AC voltages, considering that implant ingrowth is not only realised by the pre-existing osteoblasts but also by the immigrating precursor cells that undergo differentiation into the osteogenic lineage. The influence of AC on mesenchymal stem cells has been investigated by Hronik-Tupaj et al. [28]. In this previous study, the human bone marrow-derived stem cells were exposed to a 2 V/m AC field with a frequency of 60 kHz for 40 min per day [28]. The researchers found an increase in *COL1A1* and *ALP* transcripts on day 15 and 20, which were associated with an increase in heat shock protein 27 transcripts and a higher metabolic activity as compared to the unstimulated controls. However, collagen fibres and calcium deposition were not increased in stimulated samples, compared to the control samples [28]. McCullen et al. stimulated adipose-derived stem cells with very high AC fields ranging from 1–1000 V/cm at 1 Hz frequency for four hours per day [29]. The cell viability and attachment were maintained at a maximum of 10 V/cm. While 1 V/cm increased the calcium deposition as compared to the controls on day 28, the rise in intracellular calcium was first seen when 10 or 100 V/cm was applied [29].

Further, the substrate in which the cells are electrically stimulated by ES is important for the ES effects. In a previous study, human osteoblasts cultured on three-dimensional collagen scaffolds and stimulated with a magnetic field and an additional alternating electric field strongly increased the collagen synthesis [30]. The effects could be due to a coupled mechanical stimulation induced by the piezoelectric properties of the collagen scaffold [31]. However, the osteoblast function was also increased during the electromagnetic stimulation when cultured in a non-piezoelectric three-dimensional matrix using a similar test set-up [32]. Jin and Kim exposed the osteoblast-like MG-63 cells to AC fields (5.5 V/m, 60 Hz, 30 min per day) during culture on a different polycaprolactone (PCL)-based scaffold [33]. They found elevated ALP activity and calcium deposition after ES on day 14, with the highest effects apparent in MG-63 cells seeded on PCL scaffolds that included β-tricalcium phosphate [33]. The conductive polypyrrole (PPY)/PCL scaffold used by Zhang et al. led to an increased cell proliferation compared to pure PCL scaffolds, with both scaffolds under electrical stimulation (0–250 μA, DC) [34]. In our present study, we stimulated the human osteoblasts on collagen-coated coverslips to supply a physiologic surface. Three-dimensional conductive scaffolds, such as bioceramic scaffolds [35,36], conductive hydrogels [37], or biodegradable polymers as polyaniline scaffolds [38], can be implemented in further studies to approach better physiologic conditions in order to adjust the electrical field distribution during electrical stimulation. A limitation of our present study is that the complex in vivo situation, including the interplay of different bone and precursor cells interacting via cell contacts and various cytokines and growth factors, has not been considered. Therefore, a co-cultivation of different osteogenic cells, like osteoblasts, osteoclasts, and bone marrow stem cells, should be applied in future experiments to understand the interplay between different cell types under ES. Additionally, scaffolds can be used to not only mimic the three-dimensional tissue structure but also reflect the possible replacement material which can be used in surgery.

## 4. Materials and Methods

### 4.1. Isolation and Cultivation of Human Osteoblasts

Human primary osteoblasts were isolated from the femoral heads of patients undergoing primary total hip replacement under sterile conditions, as previously described [39]. The samples were collected with the consent of the patients, following an approval by the Local Ethical Committee (Registration number: A 2010-0010, approval date: 27 January 2017). The donor cells used in the experiments were obtained from 26 females (age: 69.1 ± 11.8 years) and 25 males (age: 66.0 ± 9.9 years).

Isolated cells were cultured in Dulbecco’s Modified Eagle Medium (DMEM, PAN-Biotech, Aidenbach, Germany), containing 10% fetal calf serum (FCS, PAN-Biotech, Aidenbach, Germany), 1% amphotericin B, 1% penicillin-streptomycin, and 1% Hepes buffer (all: Sigma-Aldrich, Munich, Germany) under standard cell culture conditions (5% CO_2_ and 37 °C). Ascorbic acid (final concentration: 50 µg/mL), β-glycerophosphate (final concentration: 10 mM), and dexamethasone (final concentration: 100 nM) (all: Sigma-Aldrich, Munich, Germany) were added to the cell culture medium to promote osteogenic differentiation. For the cell experiments, osteoblasts in passage three were seeded on rat tail collagen coated coverslips (diameter: 12 mm, Neuvitro, Vancouver, WA, USA) with a density of 2.2 × 10^4^ cells/cm^2^ and on Ti6Al4V electrodes with a density of 3.0 × 10^4^ cells/cm^2^ due to the roughness and the enlarged surface of the electrodes. To survey the actin staining in single cells, osteoblasts were also seeded in a lower density (i.e., 4.4 × 10^3^ cells/cm^2^ on Ti6Al4V electrodes and 1.5 × 10^4^ cells/cm^2^ on collagen-coated coverslips) to ensure a closer observation of the actin cytoskeleton and a good staining quality. After adhering for 30 min at room temperature, 30 mL of cell culture medium containing osteogenic additives were added, and the stimulation system was incubated under standard cell culture conditions for 12 h prior to electrical stimulation.

### 4.2. Electrical Stimulation Protocol

The in vitro stimulation system for the application of the AC voltage was designed according to the Asnis IIIs screw system used for in vivo electrical stimulation of femoral heads in humans [40]. The structure of the in vitro system and the resulting field distribution in the chamber have been described previously [25]. The electrical stimulation (ES) was started 12 h after the cell seeding. Different electrical fields were induced with 0.2, 1.4, or 2.8 V root mean square voltages, with a frequency of 20 Hz as a sinusoidal signal. The resulting electrical fields with a maximum of 1.4 V/m, 17 V/m, and 41 V/m were determined as previously described [25]. The ES was carried out using a Metrix GX 305 and GX310 function generator (Metrix Electronics, Bramley, Hampshire, UK). The AC voltage was applied three times a day for 45 min with 225 min breaks between stimulations (0.2 V, 1.4 V, 2.8 V), or continuously with 1.4 V (1.4 V cont.) for a total period of one, three, or seven days. Cells were cultivated under standard cell culture conditions. For unstimulated controls, chambers were similarly prepared without a connection to the function generator.

### 4.3. Actin Staining

Actin cytoskeleton was stained to evaluate the cell morphology, stress fibre formation, and orientation in order to exclude harmful effects of ES. Osteoblasts were washed with phosphate buffered saline (PBS, Merck KGaA, Darmstadt, Germany) and fixed in 4% paraformaldehyde for 10 min at room temperature (RT). Cells were washed in PBS and incubated with 0.5% Triton-X (Merck KGaA, Darmstadt, Germany) in PBS for five minutes at RT for permeabilisation. Afterwards, the osteoblasts were rinsed with PBS and incubated with 100 nM Acti-stain 488 fluorescent phalloidin (Cytoskeleton, Denver, CO, USA) for 30 min at RT, protected from light. The osteoblasts were washed three times with PBS, and the cell nuclei were stained with diamidino-2-phenylindole dihydrochloride (DAPI, Merck KGaA, Darmstadt, Germany) for 5 min. The images were captured with a Leica DMI 6000 (Leica Microsystems, Wetzlar, Germany) with 200× magnification.

### 4.4. Cell Proliferation

The cell numbers were quantified using DAPI, as described above, or Hoechst 33342 reagent (Thermo Fisher Scientific, Waltham, MA, USA). For the staining with Hoechst 33342, the reagent was diluted in culture medium in a concentration of 8 mg/L and cells were incubated 15 min in the dark under standard cell culture conditions. The images were captured with a Leica DMI 6000 (Leica Microsystems, Wetzlar, Germany) with 200× magnification and evaluated with the open source ImageJ software.

### 4.5. Gene Expression Analysis

For the isolation of total RNA, the osteoblasts were lysed in TriReagent^®^ (Zymo Research, Freiburg, Germany) and the samples were stored at −70 °C. Total RNA was extracted using Direct-zol™ RNA MiniPrep Kit (Zymo Research) according to the manufacturer’s instructions. Next, 50 ng of RNA from cells stimulated for one day or 100 ng of RNA from cells stimulated three and seven days were used for the cDNA synthesis with a High Capacity cDNA Reverse Transcription Kit (Applied Biosystems, Forster City, CA, USA). Semi-quantitative real-time polymerase chain reaction (PCR) for collagen type 1 (*COL1A1,* forward primer: 5′-ACGAAGACATCCCACCAATC-3′, reverse primer: 5′-AGATCACGTCATCGCACAAC-3′), alkaline phosphatase (*ALP,* forward primer: 5′-CATTGTGACCACCACGAGAG-3′, reverse primer: 5′-CCATGATCACGTCAATGTCC-3′), and osteocalcin (*BGLAP,* forward primer: 5′-TCAGCCAACTCGTCACAGTC-3′, reverse primer: 5′-GGTGCAGCCTTTGTGTCC-3′) was performed in triplicates using innuMIX qPCR MasterMix SyGreen (Analytik Jena AG, Jena, Germany). Ct-values were normalised to the house-keeping gene hypoxanthine guanine phosphoribosyl transferase (*HPRT,* forward primer: 5′-CCCTGGCGTCGTGATTAGTG-3′, reverse primer: 5′-TCGAGCAAGACGTTCAGTCC-3′) and analysed by the 2^−ΔΔct^ method [41]. The resulting gene expression is presented as the 2^−ΔΔCt^ value.

### 4.6. Collagen Type I Synthesis

The collagen type I (COL I) synthesis was investigated by the detection of the C-terminal propeptide of collagen type I (C1CP), which is released into the supernatant and directly correlates to the COL I protein biosynthesis. Supernatants containing propeptides released from osteoblasts while growing on electrodes and coverslips were collected after the experiments and stored at −20 °C until the analysis by an enzyme-linked immunosorbent assay (ELISA) (MicroVue™ CICP EIA, QUIDEL, Quidel Corporation, San Diego, CA, USA). The supernatants were treated according to the manufacturer’s instructions, and the absorbance was measured at a wavelength of 405 nm using Opsys MR microplate reader (Dynex Technologies, Denkendorf, Germany).

### 4.7. ALP Activity

For quantifying the alkaline phosphatase activity (ALP), the cells were washed with tris buffered saline two times and lysed in distilled water containing 1% Triton-X and 1% phenylmethylsulfonyl fluoride (PMSF) for 10 min at RT. The cell lysates were incubated with 1 mM p-nitrophenyl phosphate, 100 mM 2-amino-2-methyl-1-propanol, and 5 mM MgCl_2_ in distilled water for one hour at 37 °C, and the reaction was stopped with 2 M NaOH. Absorbance of the solution was detected at 405 nm in a microplate reader (Tecan, Maennedorf, Switzerland).

### 4.8. Data Illustration and Statistical Analysis

Each test was conducted with osteoblasts obtained from at least three and up to twelve patients. The data are depicted as box plots showing the medians, 25th and 75th percentile, and the minimum and the maximum. For quantification of the cell proliferation, floating bars depicting the minimum, median, and the maximum value were used because of the small sample number (n = 3). Data from the samples exposed to the ES were compared to the unstimulated controls and are depicted as a fold change. Statistical testing was done with GraphPad Prism 7 (GraphPad Software, La Jolla, CA, USA). Differences to the controls were statistically analysed by Wilcoxon matched pairs test using raw data. For gene expression, delta Ct values were compared using Wilcoxon matched pairs test and two-way ANOVA. Differences between the different ES groups and time points were analysed by two-way ANOVA using data normalised to the controls. The level of significance was set to *p* < 0.05.

## 5. Conclusions

Our in vitro system exclusively enables the electrical stimulation of bone cells growing on a model implant surface, thereby delivering electrical stimulation of the electrodes and the surroundings in one experiment. Suitable and inappropriate AC voltages for low-frequency bone stimulation were identified, as higher electrical stimulation with 2.8 V significantly reduced osteogenic differentiation factors. The efficiency of short-term ES, as opposed to continuous stimulation, has been demonstrated, supporting clinical approaches using temporary ES due to its practicability.
